# Serosurvey and associated risk factors for *Neospora* *caninum* infection in Egyptian water buffaloes (*Bubalus* *bubalis*)

**DOI:** 10.1038/s41598-023-50087-3

**Published:** 2023-12-21

**Authors:** Ayed Alshammari, Mohamed Marzok, Hattan S. Gattan, Mohamed Salem, Omar A. Al-Jabr, Abdelfattah Selim

**Affiliations:** 1 Department of Biology, College of Science, University of Hafr Al-Batin, Hafr Al-Batin, Saudi Arabia; 2 Department of Clinical Sciences, College of Veterinary Medicine, King Faisal University, 31982 Al-Ahsa, Saudi Arabia; 3 Department of Surgery, Faculty of Veterinary Medicine, Kafr El Sheikh University, Kafr El Sheikh, Egypt; 4 Department of Medical Laboratory Sciences, Faculty of Applied Medical Sciences, King Abdulaziz University, Jeddah, Saudi Arabia; 5 Special Infectious Agents Unit, King Fahad Medical Research Center, King AbdulAziz University, Jeddah, Saudi Arabia; 6 Department of Medicine and Infectious Diseases, Faculty of Veterinary Medicine, Cairo University, Cairo, 12613 Egypt; 7 Department of Microbiology, College of Veterinary Medicine, King Faisal University, P.O. Box 400, 31982 Al-Ahsa, Saudi Arabia; 8 Department of Animal Medicine (Infectious Diseases), Faculty of Veterinary Medicine, Benha University, Toukh, 13736 Egypt

**Keywords:** Bacteriology, Risk factors

## Abstract

Neosporosis is a parasitic disease that causes reproductive disorders in animals, making it a barrier to maximum efficiency. The purpose of this study was to determine the seroprevalence of *Neospora* *caninum* (*N.* *caninum*) antibodies in water buffaloes from four governorates in northern Egypt. A commercial indirect-ELISA test was used to detect antibodies against *N.* *caninum* in the serum of 450 water buffaloes. The total seroprevalence of *N.* *caninum* in water buffaloes from Egypt was 31.3%, and the highest prevalence was observed in Gharbia governorate. The identified risk factors for *N.* *caninum* infections in water buffaloes were sex (OR = 1.96, 95%CI: 1.22−4.17), buffaloes more than 4 years of age ( OR = 5.80, 95%CI: 2.26−14.86), abortion in second trimester (OR = 16.48, 95%%CI: 2.99−34.03), history of abortion (OR = 3.45, 95%CI: 1.58−7.52) and contact with dogs (OR = 2.55, 95%CI: 1.51−4.32). Thus, more studies are needed to determine the role of buffaloes in the epidemiology of neosporosis in Egypt.

## Introduction

Neosporosis is one of important cause for abortion and reproductive abnormalities in vulnerable animal around the world, resulting in significant economic losses, notably among ruminants^1–3^. It is a worldwide parasitic disease caused by *Neospora* *caninum*, protozoan apicomplexan that includes canids as definitive hosts while birds and numerous species of mammals as intermediate hosts^4,5^. The main way of *N.* *caninum* infection in ruminants is vertical transmission or transplacental^6^. In addition, the horizontal transmission is possible via consumption of tissues harboring cysts and tachyzoites as well as the consumption of sporulated oocysts in contaminated feed or water^7–9^.

This parasite is a significant contributor to bovine foetal miscarriage, stillbirth, the birth of weak calves, and lower milk supply. Moreover, due to the absence of viable options for the prevention and treatment of this disease, culling of infected animals is frequently necessary^10^. Buffalo is regarded as natural intermediate hosts of *N.* *caninum*, despite the fact that water buffalo differ from cattle in behaviour and physiology and can act as reservoirs for infectious disease agents of cattle^11^. Experiments on buffaloes have demonstrated that pregnant animals are vulnerable. However, buffaloes are more resistant to abortion, and their inflammatory reactions to infection are weaker than those of cattle^12^.

Several studies have been detected antibodies against *N.* *caninum* in water buffaloes in many countries over the world. The average seroprevalence of *N.* *caninum* in water buffaloes in worldwide is 48%^12^, which is much greater than those in beef cattle (11.5%) and in dairy cattle (16.1%)^13^. Among these, *N.* *caninum* seroprevalence in water buffalo is 20% in Asia, 54.4% in South America, and 67.1% in Africa^12^. However, few studies have been conducted to investigate the risk factors for *N.* *caninum* infection in water buffaloes. The age of buffaloes and the presence of dogs on the property are considered as risk factors for incidence of neosporosis^14,15^.

In Egypt, *N.* *caninum* have been reported in various animal species, the seroprevalence rates were ranged between 6.97% to 42.8% in water buffaloes^16^, 12.21% to 20.43% in cattle^16–18^, 79.7% in sheep^19^, 93.4% in goats^19^ and 10.9% in camels^3^. Controlling of neosporosis requires the identification of risk factors for the presence of antibodies to *N.* *caninum*, particularly in the absence of efficient immunoprophylactic or treatment regimens since potential risk factors may change between geographical locations. However, few studies have been conducted to evaluate the associated risk factors for *N.* *caninum* infection.

Consequently, the purpose of this research was to estimate the seroprevalence of *N.* *caninum* in water buffaloes in some Egyptian governorates situated at North and Nile Delta of Egypt and to identify the risk factors associated for *N.* *caninum* infection.

## Materials and methods

### Ethical statement

The Benha University ethics committee for animal experiments approved all methods including the handling and collection of blood samples. In addition, all methods were performed in accordance with the relevant guidelines and regulations. Owners of the buffaloes gave their explicit consent for the collection of the samples. The ARRIVE criteria were adhered to throughout the study process.

### Study area

**Figure 1:**
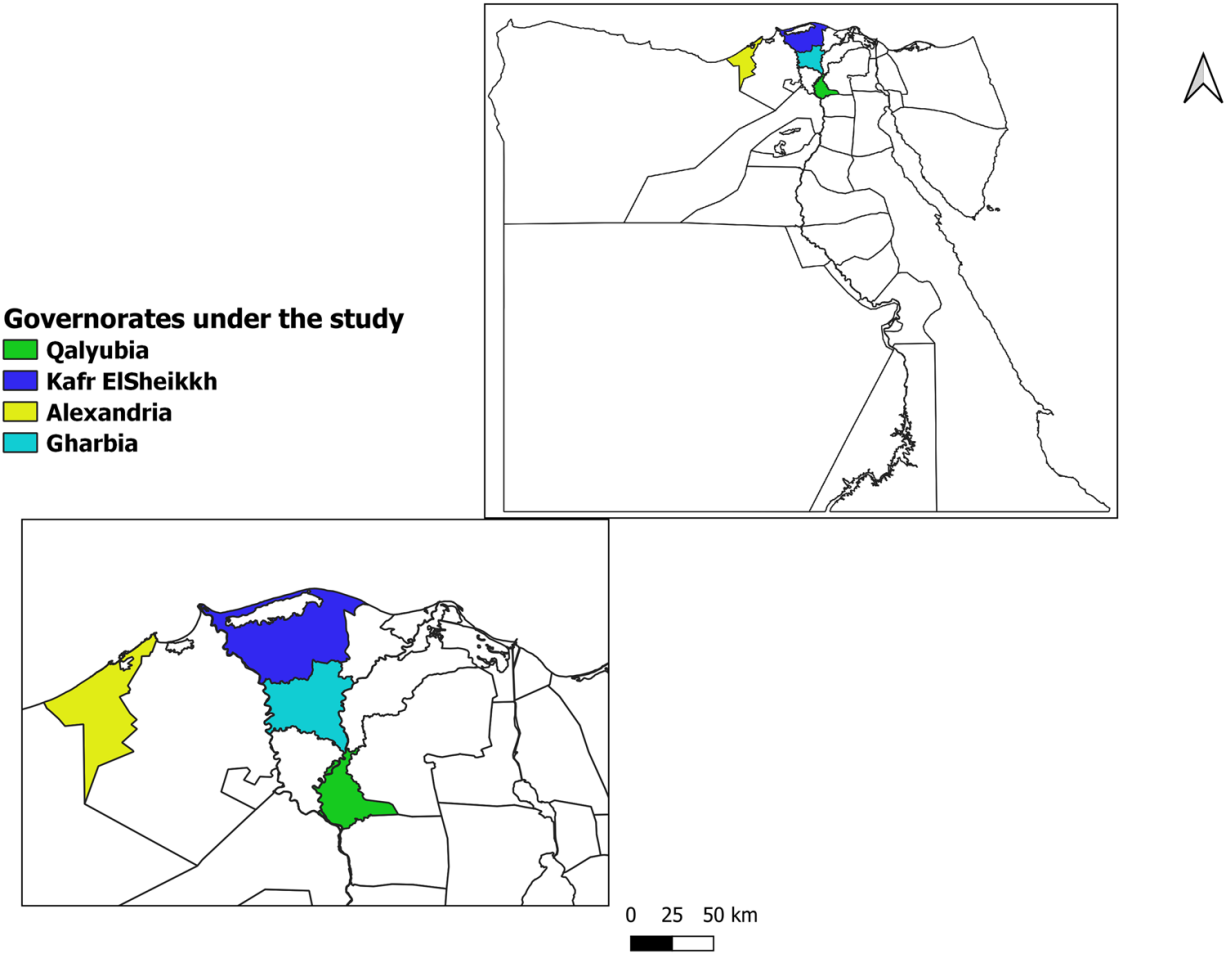
Map showed the governorates under the study (map generated by QGIS program).

For this study, the four governorates in northern Egypt with the greatest water buffalo populations were selected. The four governorates chosen for the study were Kafer ElSheikh, Qalyubia, Gharbia and Alexandria located at latitudes 31°06′42′′ N, 30.41° N, 30.867° N and 31°11′51″ N, respectively, and longitudes 30°56′45′′ E, 31.21° E, 31.028° E and 29°53′33″ E, respectively (Fig. 1).

Kafr ElSheikh, Qalyubia and Gharbia situated at Nile Delta of Egypt, which characterized by moderate weather at summer and the average annual temperature is 28 °C (with a range of 17 °C to 42 °C), and its average annual precipitation is between 100 to 200 mm. In addition, the prevailing north wind, blowing across the Mediterranean, gives Alexandria a less harsh climate than the surrounding desert.

### Study design and sample size

A cross-sectional study was performed during September 2020 to August 2021 to determine the prevalence of antibodies against *N.* *caninum* in water buffaloes in the four studied governorates. Using Epi Info™ 7, a representative sample size of 450 buffaloes was determined with a 95% confidence interval, a 5% statistical error, and a 42.8% expected seroprevalence for *N.* *caninum*^16^. A total of 450 water buffaloes of various ages and both sexes were chosen at simple random technique from individual animal raised by individual farmers. All of buffaloes included in the study were apparent healthy without in clinical signs and some of them had history of abortion.

Blood samples (5 mL) were obtained from the jugular vein, centrifuged at 3000 rpm for 10 min, serum separated, and kept at −20 °C. An epidemiology questionnaire was used at the time of sampling to collect data from each farmer on the study region, age, sex, breeding system (household or farming system), time of the abortion, previous abortions, and contact with dogs. The information about history and number of abortion or contact with dogs depends on data collected from farmers.

### Serological analysis

The antibodies against *N.* *caninum* were detected in all sera using commercial indirect ELISA Kit (IDEXX Laboratories Inc., Westbrook, USA). The sensitivity of this kit is 100%, while the specificity 98.9%. the optical density (OD) was measured at 650 nm using microplate ELISA reader (AMR-100, AllSheng, China). Serum samples considered to be positive for *N.* *caninum* if their sample to positive (S/P) ratios were more than or equal to 0.50.

### Data analysis

Data were collected and analyzed using SPSS ver. 24 program (IBM, USA). A univariate analysis of the variables of interest was carried out using the Pearson's chi-squared test to determine factors related to *N.* *caninum* infection. The result of *P*-value < 0.05 was regarded as statistically significant. The results were analysed through the use of univariable logistic regression to evaluate the association between each variable and prevalence of *N.* *caninum* in buffaloes. A logistic regression analysis was used to determine the relationship between the existence of anti-*N.* *caninum* antibodies and risk variables (sex, age, time and history of abortion, and contact with dogs). The multivariate regression model was used to assess odds ratios (ORs), and confidence intervals (CIs) of each significant variable^20,21^. Odds ratios more than one indicated an increased risk of *N.* *caninum* seroprevalence, whereas odds ratios less than one indicated a lower risk of *N.* *caninum* seroprevalence. The model's fit was evaluated using the Hosmer and Lemeshow goodness test.

## Results

We examined 450 buffalo sera from four Egyptian governorates between September 2020 and August 2021, and the findings revealed that 31.1% (141/450) of examined animals had antibodies against *N.* *caninum,* with non-significant variation (*P* = 0.238) between studied governorates, Table 1.

**Table 1:** Seroprevalence of *N.* *caninum* in water buffaloes in relation to different studied factors.

Factor	Total examined buffaloes	No of positive	No of negative	% of positive	95% CI	Statistic
Locality						
Qalyubia	110	29	81	26.4	19.03–35.29	χ^2^ = 4.224 df = 3 *P* = 0.238
Kafr ElSheikh	120	33	87	27.5	20.3–36.09	
Gharbia	100	36	64	36.0	27.27–45.76	
Alexandria	120	43	77	35.8	27.81–44.73	
Sex						
Male	85	10	75	11.8	6.51–20.31	χ^2^ = 18.651 df = 1 *P* < 0.0001*
Female	365	131	234	35.9	31.14–40.93	
Age						
< 2 years	52	9	43	17.3	9.38–29.73	χ^2^ = 33.409 df = 2 *P* < 0.001*
2–4 years	289	74	215	25.6	20.92–30.94	
> 4 years	109	58	51	53.2	43.89–62.31	
Breeding system						
Household	310	99	211	31.9	27–37.32	χ^2^ = 0.168 df = 1 *P* = 0.682
Farming	140	42	98	30.0	23.03–38.04	
Time of abortion						
First trimester	25	6	19	24.0	11.5–43.43	χ^2^ = 29.070 df = 2 *P* < 0.0001*
Second trimester	65	53	12	81.5	70.45–89.11	
Third trimester	37	17	20	45.9	31.04–61.62	
History of abortion						
Yes	127	76	51	59.8	51.15–67.96	χ^2^ = 48.563 df = 2 *P* < 0.0001*
No	323	65	258	20.1	16.11–24.83	
Contact with dogs						
Yes	280	109	171	38.9	33.41–44.75	χ^2^ = 13.213 df = 1 *P* < 0.0001*
No	170	32	138	18.8	13.66–25.36	
Total	450	141	309	31.3	27.22–35.76	

*The results considered significant if *P*-value less than 0.05.

Several factors including sex, age, breeding system, time of abortion, history of abortion and contact with dogs were analyzed to determine the risk factors related with *N.* *caninum* infection in water buffalo (Table 2).

**Table 2:** Multivariate logistic regression analysis for variables associated with seroprevalence of *N.* *caninum* in water buffaloes.

Variables	B	S.E	OR	95% CI for OR	*P* value
Sex					
Female	0.675	0.384	1.96	1.22–4.17	0.014
Age					
2–4 years	0.406	0.452	1.50	1.11–3.52	0.035
> 4 years	1.758	0.480	5.80	2.26–14.086	< 0.0001
Time of abortion					
Second trimester	0.369	0.519	16.48	2.99–34.03	< 0.0001
Third trimester	2.802	0.370	13.45	1.59–7.53	0.002
History of abortion					
Yes	1.240	0.397	3.45	1.58–7.52	0.002
Contact with dogs					
Yes	0.938	0.268	2.55	1.51–4.32	< 0.0001

*B* logistic regression coefficient, *SE* standard error, *OR* odds ratio, *CI* confidence interval.

There was significant difference (*P* < 0.05) in seroprevalence in relation to sex and age of examined water buffaloes. The seroprevalence of *N.* *caninum* was greater in females (35.9%) than males (11.8%), and in elder buffaloes over 4 years old (53.2%) as compared to young buffaloes under 2 years (17.3%) or buffaloes aged 2–4 years (25.6%). Furthermore, the seroprevalence of *N.* *caninum* was significantly (*P* < 0.05) higher in buffaloes with a history of abortion (59.8%), particularly those aborted in the second trimester (81.5%). In the current investigation, the seroprevalence of *N.* *caninum* in buffaloes kept in touch with dogs was substantially greater (38.9%) than in other animals kept away from dogs, Table 1.

Multivariable logistic regression analysis was performed to assess the risk factors associated with *N.* *caninum* infection in water buffaloes, Table 2. The results revealed that females (OR = 1.96, 95%CI: 1.22−4.17), buffaloes more than 4 years age (OR = 5.80, 95%CI: 2.26−14.86), abortion in second trimester (OR = 16.48, 95%%CI: 2.99−34.03), history of abortion (OR = 3.45, 95%CI: 1.58−7.52) and contact with dogs (OR = 2.55, 95%CI: 1.51−4.32) were identified as risk factors for *N.* *caninum* infection in water buffaloes, Table 2.

## Discussion

Infection with *N.* *caninum* causes retained fetal membranes and abortion, and subsequent infections raise the risk of reproductive disorders in water buffalo, which causes significant financial losses to the agricultural sector^5,22,23^. However, the available data on the prevalence of associated risk factors for *N.* *caninum* infections in Egyptian water buffaloes are scarce.

In the present study, the overall seroprevalence rate of *N.* *caninum* in water buffaloes was 31.3%, falling within the previously reported range (27.5% to 35.4%) in Brazil^24,25^. The reported seroprevalence rate for neosporosis in the current survey was higher than those reported in in Vietnam (1.05%)^26^, Thailand (9.1–16.7%)^27^, India (9.9%)^28^, Iran (19.3%)^29^ and Mexico (24.3–41.2%)^30,31^. Furthermore, the prevalence of the current findings appears to be lower when compared to research done in Brazil, where rates varied from 49 to 88%^32–34^, Argentina (64%)^35^ and Guangxi, China (50.9%)^36^.

The seroprevalence rates of *N.* *caninum* in buffaloes varied across countries due to a variety of factors such as climatic factors, where the mild temperature and humidity help in growth of oocyst of Neospora^5,37,38^. In addition, the rearing systems, geographic location, and farm management were varied in different countries^15,37,39–44^. Furthermore, comparing studies is challenging since researchers use varied sample criteria, serological procedures, and cut-off values, particularly for the immunofluorescence antibody test^24^.

In the line of previous results of Baltazar-Pérez, et al.^31^, the sex is significant risk factor for prevalence of *N.* *caninum* in buffaloes. Contrary to findings of Campero, et al.^35^ and Kengradomkij, et al.^27^, the results revealed strong association between *N.* *caninum* seroprevalence in water buffaloes and sex. According to our findings, vertical transmission is frequently thought to be the primary mode of infection in bovines^45^.

According to age group, adult animals in our study had a much greater prevalence than young animals. Similarly, several studies have been found significant association between age and the seropositivity for neosporosis in water buffaloes^14,46–49^. Indeed, elder age would suggest a possibly longer interaction with polluted water and/or feed, explaining the increased occurrence. It has been proposed that the age correlation merely reflects the annual increase in the likelihood of being exposed to parasite oocysts. In comparison to younger animals, older animals are predicted to have a larger likelihood of cumulative exposure to infectious agents. In contrast, the seroprevalence of *N.* *caninum* in water buffaloes from northern Brazil were not affected by age factor^33,41,50–52^. Interestingly, calves had a significant seroprevalence rate (17.3%), the persistence of maternal antibodies or trans-placental transmission^47^ might be to blame for this. According to Cardoso, et al.^53^, maternal antibodies against *N.* *caninum* can last up to 21 weeks after birth.

In contrast to the findings of Bărburaș, et al.^46^, we observed no significant relationship between breeding system and *N.* *caninum* seroprevalence. The seroprevalence rate was greater in farm animals than in house-hold animals, which was due to the chance of farm animals being exposed to sources of parasite oocyst contamination being higher.

There is no information in Egypt about buffalo reproductive losses caused by *N.* *caninum* infection. The findings of this study revealed strong association between *N.* *caninum* infection and abortion especially in second trimester stage. These findings were consistent with what have been found in previous studies of Romero-Salas, et al.^30,54^, Anderson, et al.^55^ and Wouda, et al.^56^. This provides circumstantial evidence that *N.* *caninum* may have a role in water buffaloes abortions in the research region.

Similarly, to the findings of Oliveira, et al.^24^, *N.* *caninum* prevalence rate was greater among water buffaloes kept with close contact with pet dogs. This could be attributed to dogs consuming aborted materials and consequently shedding infective oocyst which contaminate food and play a significant role in horizontal spreading of *N.* *caninum* infection to vulnerable animals^57^.

The limitation of this study is random sampling because it does truly represent about the prevalence of the disease in the studied areas.

## Conclusion

The present findings confirmed that presence of antibodies against *N.* *caninum* in water buffaloes in studied governorates in Northern Egypt. In addition, some factors such as sex, age, time or history of abortion and contact with dogs considered as potential risk factors for *N.* *caninum* infection. These findings highlight the relevance of *N.* *caninum* control and prevention in Egypt, where water buffaloes are major domestic animals, as well as the need of risk factor analysis for efficient neosporosis control in various locations. The effects of *Neospora* infections in water buffalo should be further studied, but it's also important to look at the epidemiological significance of sick buffaloes and their connection to bovine neosporosis.

## Data Availability

This article contains all of the data that was created or analyzed throughout the investigation.
